# Directed Neural Differentiation of Mouse Embryonic Stem Cells Is a Sensitive System for the Identification of Novel Hox Gene Effectors

**DOI:** 10.1371/journal.pone.0020197

**Published:** 2011-05-26

**Authors:** Myrto Bami, Vasso Episkopou, Anthony Gavalas, Mina Gouti

**Affiliations:** 1 Developmental Biology Laboratory, Biomedical Research Foundation of the Academy of Athens (BRFAA), Athens, Greece; 2 Mammalian Neurogenesis, MRC Clinical Sciences Centre, Imperial College School of Medicine, Hammersmith Hospital, London, United Kingdom; Baylor College of Medicine, United States of America

## Abstract

The evolutionarily conserved Hox family of homeodomain transcription factors plays fundamental roles in regulating cell specification along the anterior posterior axis during development of all bilaterian animals by controlling cell fate choices in a highly localized, extracellular signal and cell context dependent manner. Some studies have established downstream target genes in specific systems but their identification is insufficient to explain either the ability of *Hox* genes to direct homeotic transformations or the breadth of their patterning potential. To begin delineating *Hox* gene function in neural development we used a mouse ES cell based system that combines efficient neural differentiation with inducible Hoxb1 expression. Gene expression profiling suggested that *Hoxb1* acted as both activator and repressor in the short term but predominantly as a repressor in the long run. Activated and repressed genes segregated in distinct processes suggesting that, in the context examined, *Hoxb1* blocked differentiation while activating genes related to early developmental processes, wnt and cell surface receptor linked signal transduction and cell-to-cell communication. To further elucidate aspects of *Hoxb1* function we used loss and gain of function approaches in the mouse and chick embryos. We show that Hoxb1 acts as an activator to establish the full expression domain of *CRABPI* and *II* in rhombomere 4 and as a repressor to restrict expression of *Lhx5* and *Lhx9*. Thus the *Hoxb1* patterning activity includes the regulation of the cellular response to retinoic acid and the delay of the expression of genes that commit cells to neural differentiation. The results of this study show that ES neural differentiation and inducible *Hox* gene expression can be used as a sensitive model system to systematically identify *Hox* novel target genes, delineate their interactions with signaling pathways in dictating cell fate and define the extent of functional overlap among different *Hox* genes.

## Introduction

The evolutionarily conserved Hox family of homeodomain transcription factors plays fundamental roles in conferring regional identity and regulating cell specification along the anterior – posterior (AP) axis during development of all bilaterian animals [1], [2]. Hox genes are expressed in rather broad domains but control cell fate choices in a highly localized, extracellular signal and cell context dependent manner [3], [4], [5]. Evidence from diverse organisms suggests that Hox proteins act partly as high-level regulators dictating the expression levels of other regulatory proteins including themselves [6], [7], [8]. They also act partly as ground level regulators, or ‘realizators’, as initially proposed by Garcia-Bellido [9], fine-tuning very diverse processes such as cell adhesion, cell division rates, cell death and cell movement [10], [11], [12], [13]. Considering their numbers, the scope of their functions, the context dependence of their actions and more than thirty years devoted to their study, few *Hox* target genes have been identified. Some studies have established direct and downstream target genes in specific systems but their identification is insufficient to explain either the ability of *Hox* genes to direct homeotic transformations or the diversity of their patterning potential.

Two main general approaches have been used, a candidate target gene approach [14], [15], [16], [17], [18] and differential gene expression analysis comparing wild type (wt) tissue with tissue in which specific *Hox* gene expression has been genetically manipulated [19], [20], [21], [22]. However, the inherent bias in choosing candidate downstream targets, functional redundancy among *Hox* genes and accumulation of secondary effects in gain or loss of function genetic models present serious limitations. The elucidation of the precise roles that *Hox* genes play in cell fate specification as well as the identification of target genes and processes are key goals to deciphering the regulatory network underlying morphogenesis of the body plan. Furthermore, this may allow harnessing their patterning potential in the directed differentiation of embryonic stem (ES) cells and induced pluripotent stem (iPS) cells to specific cell types.

During development of vertebrate neural tube the combinatorial use of *Hox* gene expression and specific dorsoventral (DV) patterning cues define specific subclasses of neuronal progenitors in the developing hindbrain and spinal cord [23]. Genetic evidence suggests that *Hox* genes act as integrators of AP and DV patterning mechanisms to generate specific classes of neuronal progenitors and neurons for the appropriate AP levels of the hindbrain and the spinal cord. For example, *Hoxb1* is specifically expressed in rhombomere 4 of the developing hindbrain. The specification of this territory and subsequent generation of r4 specific neuronal progenitors and neurons depend largely on *Hoxb1* function. Disruption of the Hoxb1 gene in mice leads to transformation of the r4 territory into an r2-like state [24], [25], whereas retroviral-mediated over-expression of *Hoxb1* in r2 causes homeotic transformation of r2 to a r4-like identity in chick [26]. In the ventral region of r4, *Hoxb1* expression is responsible for the generation of facial branchiomotor neurons and the suppression of serotonergic fate specification [24], [27]. Similarly, in more posterior regions of the developing CNS, specific *Hox* genes direct the generation of distinct motor neuron (MN) subtypes at hindbrain, brachial, thoracic and lumbar regions [28], [29], [30].

To bypass limitations in delineating *Hox* gene function in neural development we modeled the role of Hox genes in neural cell fate specification using a mouse ES cell based system that affords the possibility of inducible Hoxb1 expression. Using a differentiation protocol that generates a highly homogeneous population of neural stem (NS) cells and inducible expression of *Hoxb1* we showed that timely long term induction (8 days) of the *Hoxb1* transgene in ES cell derived NS cells resulted in the specification of NS cells toward a hindbrain specific identity through the activation of a rhombomere 4-specific genetic program and the repression of anterior neural identity [31]. These effects were accompanied by specific changes in the expression of neural progenitor markers some of which suggested that *Hoxb1* mediates neural crest cell fate induction. This was subsequently verified *in vivo* [32]. Furthermore, up regulation of the known *Hoxb1* target genes, *Hoxb2*, *Hoxa2*, *EphA2* and *Phox2b* [31] suggested that this approach could be used to identify novel *Hoxb1* target genes.

Here we use this approach and microarray gene expression profiling to identify potential novel Hoxb1 target genes and processes. To compare the long and short term effects of Hoxb1 function and limit the number of potential target genes we used a short term and a long term induction protocol. To validate the approach and elucidate aspects of Hoxb1 *in vivo* function we used loss and gain of function approaches using the chick and mouse developing embryos as model systems and investigated the *in vivo* response of two up (*CRABPI, II*) and two down (*Lhx5, 9*) regulated genes in ES derived NS cells. *Hoxb1* is itself regulated by retinoic acid [33], [34] and we found intriguing the possibility that it may regulate the expression of RA signaling effectors such as *CRABPI* and *II*. On the other hand, *Lhx5* and *9* mediate neuronal differentiation [35] and their *in vivo* repression would correlate well with the finding that *Hoxb1* blocks ES derived NS cell differentiation after mitogen withdrawal [31]. Notably, these genes have not been identified as *Hoxb1* downstream target genes in other approaches [19], [20], [36] demonstrating that ES neural differentiation and Hox inducible gene expression can be used as a sensitive model system to identify novel *Hox* target genes and processes, define binding sites and elucidate the interactions of *Hox* genes and extracellular signals in dictating neural cell fate.

## Materials and Methods

### Animals

Animal studies were conducted in accordance with international guidelines and after ethical approval of the competent Veterinary Service of Athens. The Hoxb1 mouse mutants were described and genotyped as reported [24]. Fertilized chick eggs were obtained from Pindos Hellas (Ioannina, Greece) and incubated in a humidified incubator at 38°C.

### Microarray gene expression profiling

**Figure 1:**
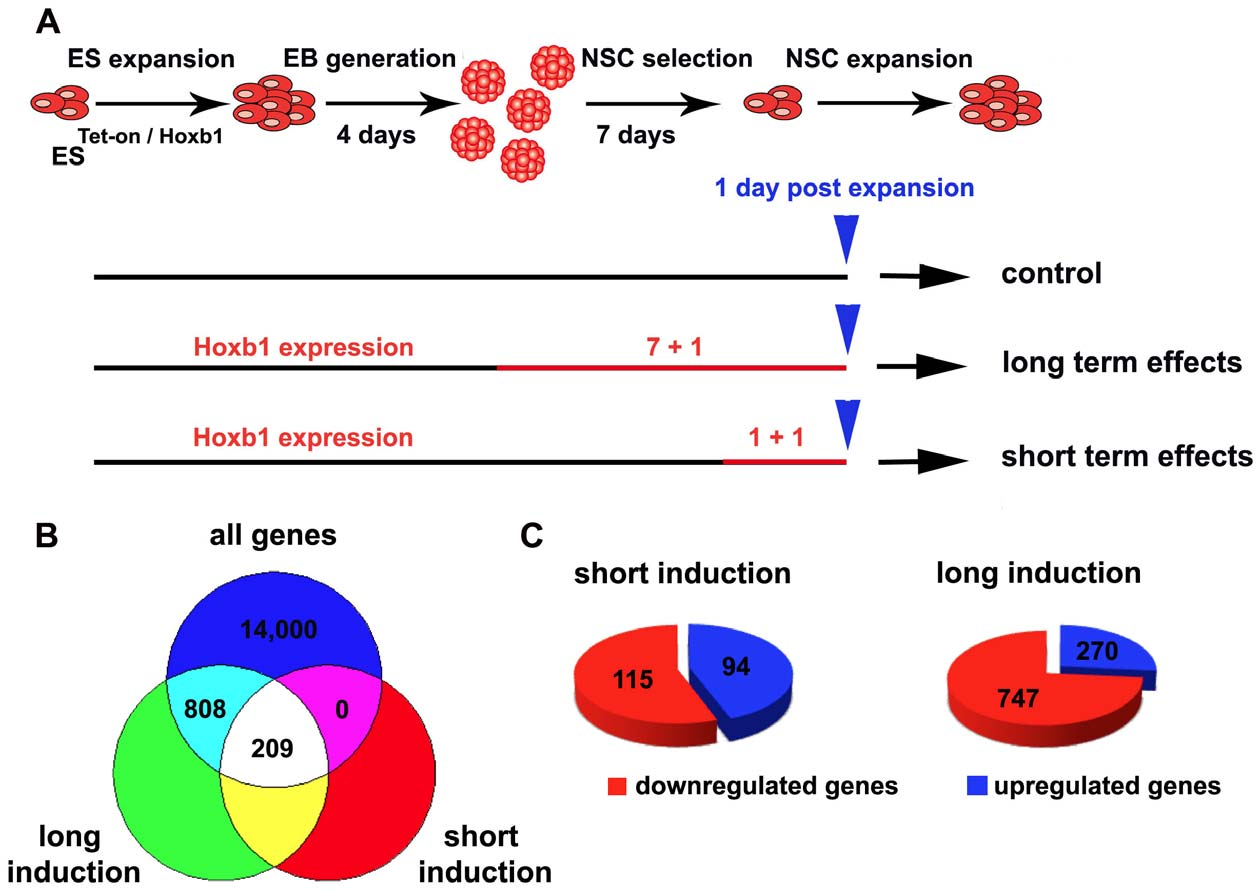
ES differentiation and Hoxb1 induction scheme, comparison of gene expression profiling results. (A) Graphic representation of ES^Tet-On/Hoxb1^ cell differentiation towards neural stem cells (NSCs) for the identification of Hoxb1 target genes. The induction length is shown in red (days) and blue arrows indicate the time point of microarray gene expression analysis. (B) Venn diagram of genes differentially regulated in the long and short *Hoxb1* induction schemes. (C) Pie charts of up and down regulated genes in the two induction schemes.

The generation and neural differentiation of the mouse ES^Tet-On/Hoxb1^ cells were as described previously [31]. For the short Hoxb1 induction scheme doxycycline (dox) was added during the last day of the selection period and for one additional day during the expansion stage (Fig. 1A). Gene expression profiling was carried out for biological triplicates for both dox induced (Hoxb1^+^) and uninduced (Hoxb1^−^) cells as described earlier [31] and the Affymetrix Mouse Genome 430A array was used. Microarray data are deposited in the public access Array Express database (Experiment ID E-MIMR-441). The list of regulated genes for the short induction scheme was restricted to genes with 0.75> fold regulation >1.3 and genes that were also present in the long induction scheme.

### Reverse transcription and Q-PCR

Total RNA was isolated from ES derived NS cells using the RNeasy kit (Qiagen) according to the manufacturer's instructions and digested by RQ1 DNase (Promega) to remove genomic DNA. First strand cDNA synthesis was performed with Superscript II reverse transcriptase (Invitrogen) using random primers. Real time PCR analysis was carried out in a Chromo4 DNA engine (Biorad), running the following program: 95°C for 10 min, then 40 cycles of 95°C for 15 s, 60°C for 40 s, followed by plate read. PCR reactions included 1x SYBR greener PCR master mix (Invitrogen), 200 nM primer and 2 ul of template in a 25 ul reaction volume. Primers were as follows (5′ to 3′):

*CRABPI* F:GGAGATCAACTTCAAGGTCGGAG,

*CRABPI* R: ATACTCCTCAGGGGAACTCGCATC,

*CRABPII* F: ACATCAAAACCTCCACCACTGTGCGAAC,

*CRABPII* R: CGTCATCTGCTGTCATTGTCAGGATCAGC,

*Lhx5* F: GACAAGGAAACCGCTAACAACG,

*Lhx5 R:*GTGGACCCCAACATCTCAGACTCG,

*Lhx9* F: TACTTCAATGGCACTGGCACCG,

*Lhx9* R: TCCTTGGCATCTGGGTTATGG.

### *In situ* hybridization and immunofluorescence

For *in situ* hybridization embryos were fixed overnight at 4°C in 4% paraformaldehyde (PFA) in 0.1 M phosphate buffer saline (PBS). In situ hybridization was performed in whole embryos using probes for mouse *CRABPI* and *CRABPII* [37], mouse *Lhx9* [38] and for chick *Lhx9* [39] and *Lhx5* [40]. Antisense digoxigenin-labelled riboprobes were synthesized from linearized templates by the incorporation of digoxigenin-labelled UTP (Boehringer) using T3 or T7 polymerase. Processing of the embryos and hybridization with 500 ng/ml of the probe was as described previously [25]. After whole mount *in situ* hybridization, embryos were fixed again overnight at 4°C and then processed for immunofluorescence. For immunofluorescence embryos were fixed in 4% PFA in PBS for 1–2 h at 4°. Embryos were cryoprotected with 30% sucrose in PBS and cryosectioned. Blocking was carried out in 10% normal goat serum (NGS) with 0.1% triton for 1 h at RT. The cryosections were incubated overnight at 4°C with the primary antibody diluted in 1% NGS, 0.1% triton in PBS. Primary antibodies used were as follows: rabbit anti-Hoxb1, 1∶400 (Covance), mouse Lhx5, 1∶100. Secondary antibodies were anti-mouse and anti-rabbit Alexa 488 or Alexa 568 (Molecular Probes) used at 1∶500. Images were acquired using a Leica TCS SP5 confocal microscope.

### Chick in ovo electroporation

Chick embryos were staged according to Hamburger and Hamilton (HH) (Hamburger and Hamilton, 1951) and electroporated at HH stage 10–11. Chick embryos were electroporated with plasmid DNA at a concentration of 1.5 µg/µl. The coding regions of mouse *Hoxb1* cDNA was inserted into the pCAGGS-IRES-NLS-GFP expression vector [41] upstream of the IRES. As a control, pCAGGS-IRES-NLS-GFP was included at 0.5 µg/µl. Electroporation was carried out using a BTX ECM830 electroporator delivering five 20 V pulses of 50 millisecond duration each. Electroporated embryos were dissected at the desired stage and fixed for *in situ* hybridization or immunofluorescence.

## Results

### Identification of Hoxb1 target genes

To identify potential *Hoxb1* target genes and processes we used the stable line ES^Tet-On/Hoxb1^ that allows for tight dox mediated inducible expression of the *Hoxb1* transgene at both the ES cell and NS cell stages. However, inducible expression of the transgene could mobilize the endogenous *Hoxb1* autoregulatory loop only at the NS cell stage demonstrating the importance of cellular context for *Hoxb1* function and its analysis. *Hoxb1* induction using an 8-day long dox exposure resulted in the generation of r4 specific neuronal progenitors [31]. Microarray gene expression analysis was used to identify the genes that were regulated at the end of that period (Table S1). To reduce the number of likely *Hoxb1* downstream effectors and compare the short term and long term effects of *Hoxb1* expression we performed microarray gene expression analysis after a two day long exposure to dox (Fig. 1A). Analysis of the microarray data using fold regulation cut offs (0.75< fold regulation >1.3) and stringent statistical criteria (FDR <0.005) showed that the number of regulated probe sets increased with time from 209 regulated genes at 2 days of exposure to 1017 regulated genes at 8 days of exposure (Fig. 1B, Table S1 and Table S2). Interestingly, the percentage of repressed genes increased from 55% to 73% with time suggesting that the long-term effects of Hoxb1 expression were primarily to repress genes and thus exclude alternative fates, consistent with *Hoxb1* acting as a cell fate selector gene (Fig. 1C). To identify Hoxb1 regulated processes we performed Gene Ontology (GO) analyses for the genes identified in the long-term induction scheme. Strikingly, repressed and activated genes segregated in distinct GO processes. Up regulated genes were associated with early patterning and developmental activities including signaling whereas down regulated genes were associated with late, differentiation processes (Table 1).

**Table 1:** Hoxb1-regulated biological processes.

	GO ANALYSIS	RATIO	p VALUE	p VALUE
			DOWNREGULATED	UPREGULATED
**1**	GO:48731: system development	131/1153	**7,05e-12**	0,00061
**2**	GO:7399: nervous system development	123/1089	**4,75e-11**	0,00106
**3**	GO:30182: neuron differentiation	63/493	**3,54e-8**	0,172
**4**	GO:30154: cell differentiation	1681811	**5,11e-8**	0,00244
**5**	GO:7409: axonogenesis	40/273	**3,36e-7**	0,752
**6**	GO:48468: cell development	72/639	**5,96e-7**	0,433
**7**	GO:48667: neuron morphogenesis during differentiation	43/326	**2,23e-6**	0,689
**8**	GO:904: cellular morphogenesis during differentiation	46/364	**3,28e-6**	0,768
**9**	GO:902: cellular morphogenesis	88/872	**3,82e-6**	0,187
**10**	GO:7417: central nervous system development	36/259	**4,43e-6**	0,08
**11**	GO:48666: neuron development	49/403	**4,74e-6**	0,519
**12**	GO:9966: regulation of signal transduction	45/387	**3,49e-5**	0,479
**13**	GO:7420: brain development	28/202	**5,16e-5**	0,0268
**1**	GO:9653: morphogenesis	155/1903	0,000207	**5,41e-9**
**2**	GO:48513: organ development	131/1893	0,0904	**6,85e-8**
**3**	GO:9790: embryonic development	34/554	0,542	**5,07e-7**
**4**	GO:9887: organ morphogenesis	65/989	0,318	**8,95e-7**
**5**	GO:16055: Wnt receptor signaling pathway	23/227	0,0136	**5,53e-6**
**6**	GO:7166: cell surface receptor linked signal transduction	150/2295	0,239	**2,78e-5**
**7**	GO:7154: cell communication	422/5965	0,000593	**7,74e-5**

After gene expression profiling of cells in the long induction scheme upregulated and downregulated genes were separately subjected to GO analysis. The ratio is represented by the number of genes regulated in a particular GO category over the total number of genes in that GO category.

**Figure 2:**
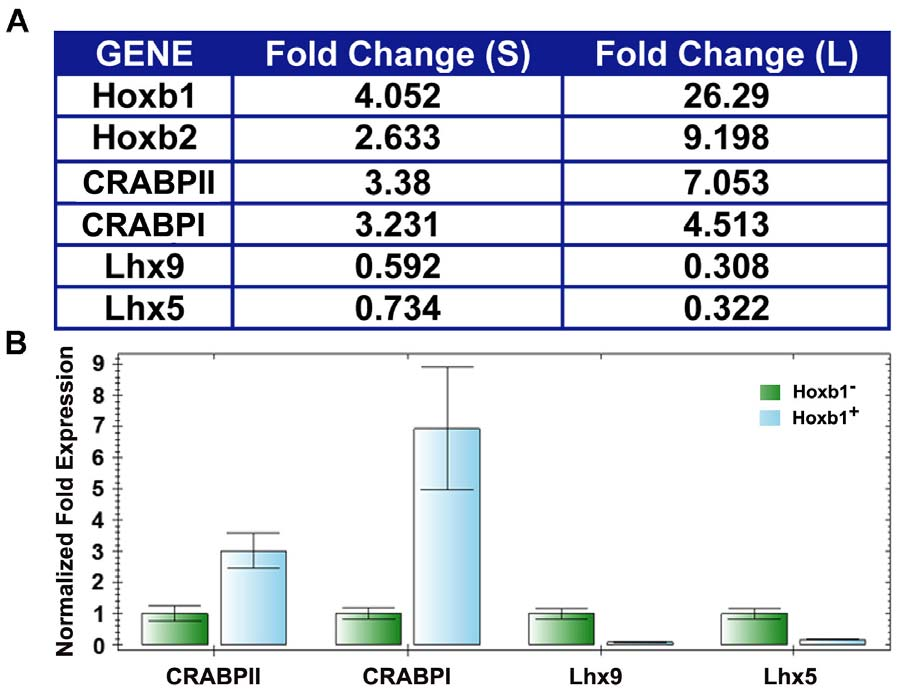
Hoxb1 regulation of selected genes validated by RT-PCR. (A) Hoxb1 mediated fold regulation of *CRABPI*, *CRABPII* and *Lhx5* and *Lhx9* expression in the short (s) and long (l) induction schemes. As a comparison, the regulation of two know Hoxb1 targets, *Hoxb1* itself and *Hoxb2* is shown. (B) Real – time PCR confirmation of differences in the expression of *CRABPI* and *II* and *Lhx9* and *5* in Hoxb1^−^ and Hoxb1^+^ cells.

We then turned to choosing genes for *in vivo* validation. To increase specificity, we focused on genes regulated in both short and long term exposure experiments (Table 2). Regulation was towards the same direction with the notable expression of only three genes and generally stronger in the long term (for a full list see Table S2). We then used qPCR and *in vivo* loss and gain of function approaches to validate the results for two up regulated and two down regulated genes. Real Time PCR analyses for the regulation of *CRABPI*, *CRABPII*, *Lhx5* and *Lhx9* using the long induction scheme yielded results that were in good agreement with the microarray results (Fig. 2) suggesting that they were appropriate candidates for further, *in vivo* analyses.

**Table 2:** Hoxb1 regulated genes.

Description	Gene Symbol	Fold Change (s)	Fold Change (l)
homeo box B1	Hoxb1	4.052	26.29
homeo box B2	Hoxb2	2.633	9.198
parathyroid hormone-like peptide	Pthlh	2.49	7.467
cellular retinoic acid binding protein II	Crabp2	3.38	7.053
LIM homeobox protein 8	Lhx8	1.578	6.477
chemokine (C-X-C motif) ligand 14	Cxcl14	1.407	5.723
gamma-aminobutyric acid receptor, subunit gamma 1	Gabrg1	2.432	5.25
leucine-rich repeat LGI family, member 2	Lgi2	1.353	5.14
procollagen, type XIV, alpha 1	Col14a1	1.954	4.731
steroid 5 alpha-reductase 2-like 2	Srd5a2l2	2.741	4.585
cellular retinoic acid binding protein I	Crabp1	3.231	4.513
ret proto-oncogene	Ret	1.911	4.382
aldolase 3, C isoform	Aldoc	1.451	4.213
T-cell lymphoma invasion and metastasis 2	Tiam2	1.327	3.526
solute carrier family 18, member 3	Slc18a3	2.604	3.383
aldo-keto reductase family 1, member C12	Akr1c12	1.589	3.295
claudin 11	Cldn11	1.497	3.2
LIM homeobox protein 5	Lhx5	0.734	0.322
cerebellin 1 precursor protein	Cbln1	0.461	0.322
forkhead box G1	Foxg1	0.565	0.283
wingless-related MMTV integration site 7B	Wnt7b	0.625	0.28
LIM homeobox protein 2	Lhx2	0.676	0.277
OTU domain containing 1	Otud1	0.666	0.265
LIM homeobox protein 9	Lhx9	0.539	0.215
R-spondin 2 homolog (Xenopus laevis)	Rspo2	0.567	0.214

List of genes regulated in both short (s) and long (l) induction schemes with False Discovery Rate (FDR) <0.005.

### Hoxb1 modulates RA signaling by regulating expression of *CRABPI* and *CRABPII* in r4

The results presented above suggested that Hoxb1 patterns the hindbrain at least partly by modulating the cellular response to RA through the regulation of *CRABPI* and *CRABPII*. To examine this hypothesis we compared the *CRABPI* and *CRABPII* expression in *wt* and *Hoxb1^−/−^* mouse embryos at 10.5 dpc using *in situ* hybridization.

**Figure 3:**
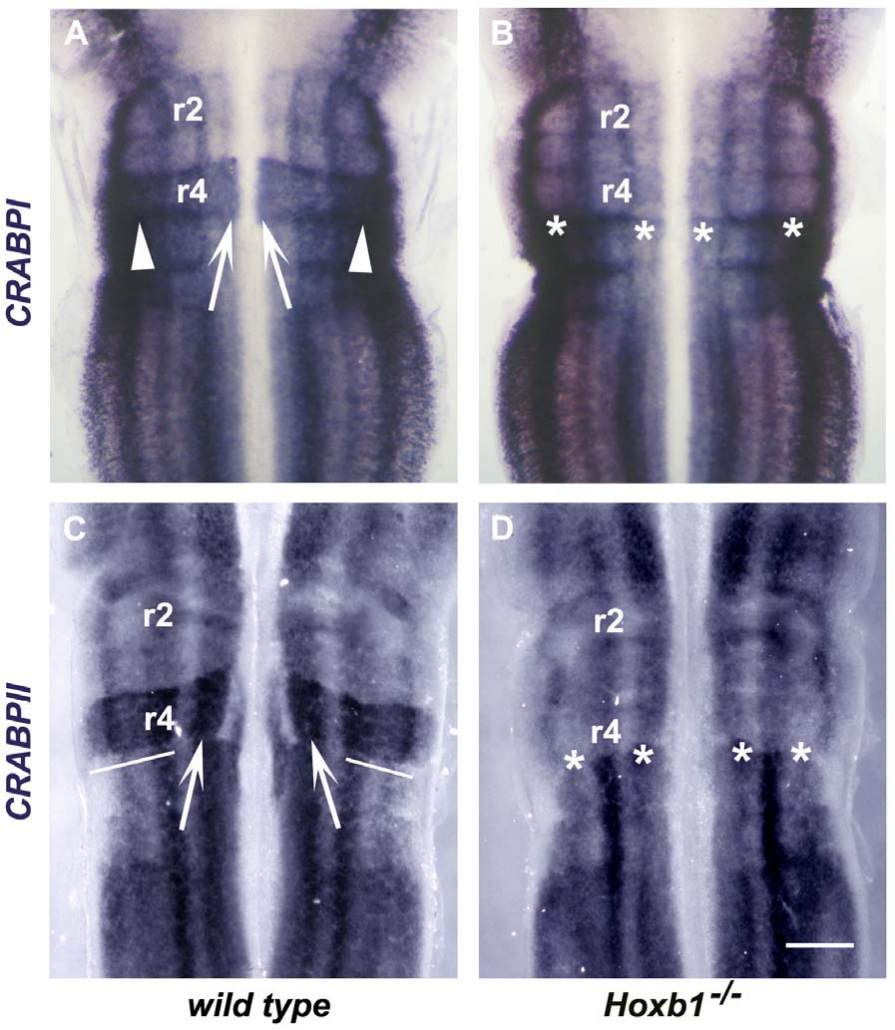
Expression of CRABPI and CRABPII in the hindbrain of wt and Hoxb1^−/−^ mouse embryos at 10.5 dpc. (A – D) Ventricular views of flat mounted wt (A, C) and *Hoxb1* ^−/−^ (B, D) mouse hindbrains stained with a *CRABPI* riboprobe (A, B) and a *CRABPII* riboprobe (C, D) at 10.5 dpc. r4-specific expression is denoted by arrows (A, C), arrowheads (A) and brackets (C) in wt hindbrains. r4-specific expression is lost in *Hoxb1* ^−/−^ hindbrains and denoted by asterisks (B, D). Scale bar corresponds to 450 µm.

*CRABPI* and *CRABPII* are both expressed in the developing hindbrain in a rhombomere specific manner. *CRABPI* expression first appears at the five-somite stage caudal to the preotic sulcus. During subsequent stages, expression spreads to the rest of the hindbrain but remains stronger in the caudal hindbrain, particularly in r4, 5 and 6 [37] (Fig. 3A). *CRABPII* expression appears at the same early stage as *CRABPI* in the post-otic region of the hindbrain and its expression subsequently spreads to the rest of the hindbrain [37]. *CRABPI* and *CRABPII* expression is generally stronger in r4 and the caudal hindbrain. At 10.5 dpc neural progenitors acquire specific identity and both *CRABPI* and *II* are expressed in rhombomere specific longitudinal stripes prefiguring sites of generation and differentiation of defined neuronal subtypes (Fig. 3A, C). In *wt* r4, strong *CRABPI* expression extends to a ventral domain corresponding to the resident site of facial motor neuron progenitors (arrows, Fig. 3A). Compared to more anterior rhombomeres, there is also stronger expression of *CRABPI* in dorsomedial positions of r4 (arrowheads, Fig. 3A). In *wt* r4, *CRABPI* expression is excluded from the resident site of facial motor neuron progenitors but there is strong expression in an adjacent domain (arrows, Fig. 3C) as well as in medial and dorsomedial positions of r4 (brackets, Fig. 3C).

The r4 expression pattern of *CRABPI* and *II* in *Hoxb1^−/−^* embryos changed dramatically. The ventral most expression domain of *CRABPI* was lost and expression of both *CRABPI* and *CRABPII* in medial and dorsal stripes was either lost or weakened (asterisks, Fig. 3B, D). Overall, consistent with an r4 to r2 homeotic transformation [24], [42], the r4 expression patterns of *CRABPI* and *CRABPII* in the Hoxb1^−/−^ embryos became identical to those of r2.

Thus the identification of *CRABPI* and *II* as Hoxb1 downstream genes in our screen suggested that part of Hoxb1 patterning activity may be mediated by regulation of the RA signaling activity through the up regulation of *CRABPI* and *CRABPII* gene expression.

### Hoxb1 represses the expression of *Lhx5* and *Lhx9*

**Figure 4:**
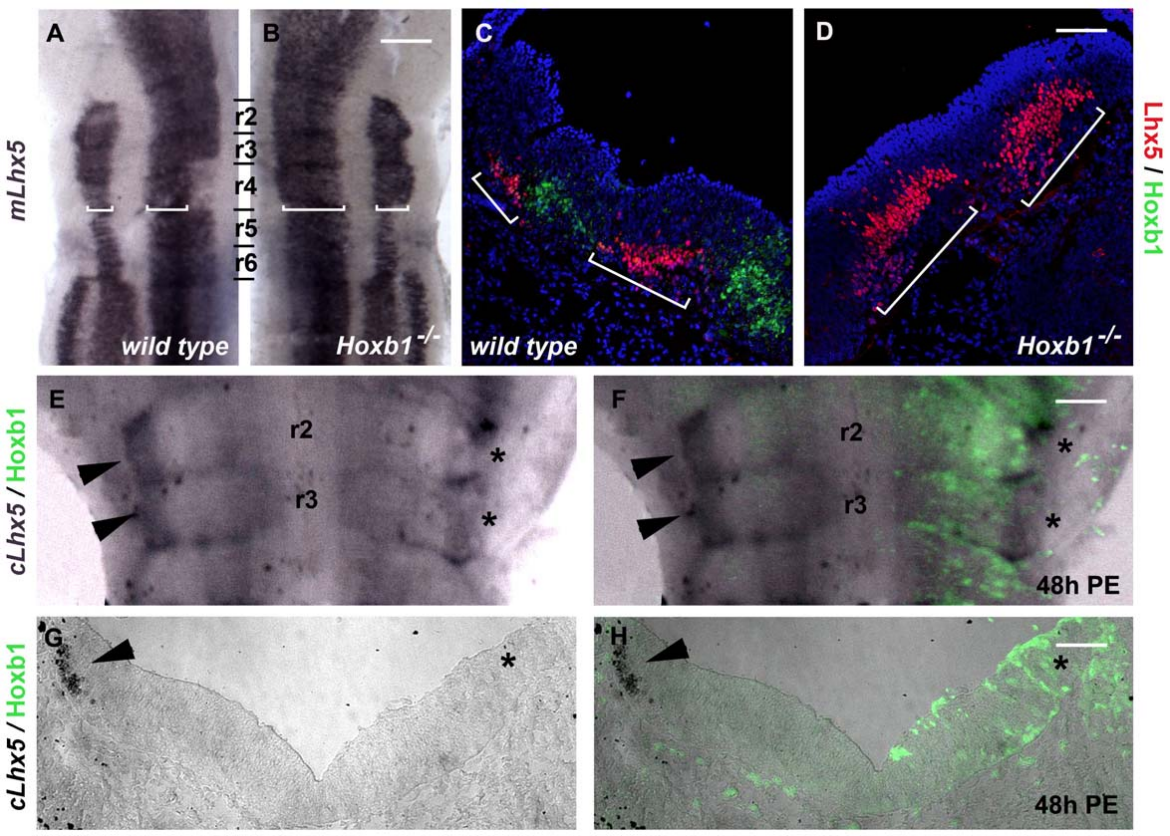
Expression of *Lhx5* in mouse and chick hindbrain after *Hoxb1* loss and gain of function experiments, respectively. (A–C) Expression of *Lhx5* in ventricular views of flat mounted hindbrains (A, B) and r4 transverse sections (C, D) using *Lhx5 in situ* hybridization alone (A, B) or in combination with Hoxb1 immunofluorescence (C, D) of wt (A, C) and *Hoxb1* ^−/−^ (B, D) 10.5 dpc embryos. *Lhx5* is expressed in two characteristic stripes in the mantle layer of r4 (A, C denoted by brackets) that expand substantially in the absence of Hoxb1 (brackets in B, D). (E–H) Expression of *Lhx5* in flat hindbrains (E, F) and r2 transverse sections (G, H) of chick embryos electroporated at stage HH 10–11 and analyzed 48 h PE by in situ hybridization for chick Lhx5 and immunofluorescence for Hoxb1 (E–H). Expression of *Lhx5* in the non-electroporated side is restricted at two dorsomedial r2 and r3 stripes (arrowheads E–H) and this expression is abolished upon Hoxb1 electroporation (asterisks E–H). Scale bar corresponds to 325 µm in A, B, to 100 µm in C, D, G, H and to 125 µm in E, F.

We then examined whether Hoxb1 can repress *Lhx5* and *Lhx9* expression *in vivo*. To study the expression of *Lhx5* in the mouse hindbrain and specifically in r4 we performed whole mount *in situ* hybridization using a specific *Lhx5* probe [38]. At 10.5 dpc in the hindbrain, *Lhx5* is expressed in two dorsoventral stripes along r1–r6 in a rhombomere specific pattern. In wt r4 there is a paucity of *Lhx5* expression in the ventral domain corresponding to the site of motor neuron progenitors whereas expression in the dorsal stripe is weaker compared to that of r2 and r3 and similar to that of r5 and r6 (brackets, Fig. 4A). In Hoxb1^−/−^ r4 *Lhx5* expression increases in both the dorsal and ventral domains and becomes similar with the expression pattern of r2 and r3 (brackets, Fig. 4B). Thus r4 expression of *Hoxb1* and *Lhx5* appeared to be mutually exclusive. This was confirmed, by Lhx5 and Hoxb1 immunofluorescence on wt r4 transverse sections (Fig. 4C). In Hoxb1^−/−^ r4 expression of Lhx5 expanded in both ventral and dorsal expression domains. This was consistent with the *in situ* hybridization results and suggested that *Hoxb1* may repress expression of *Lhx5*. To address this, we ectopically expressed *Hoxb1* in the hindbrain of HH stage 10–11 chick embryos using *in ovo* electroporation. The embryos were analyzed 48 h post electroporation (PE) (HH stage 20) by whole mount *in situ* hybridization with the chick *Lhx5 in situ* hybridization probe [40] and Hoxb1 immunofluorescence. The *cLxh5* at HH is expressed in two dorsomedial stripes in r2 and r3 (arrowheads Fig. 4E). Expression of *cLhx5* was specifically down regulated in the areas where Hoxb1 was ectopically expressed (asterisks, Fig. 4E, F) and this was confirmed by r2 transverse sections showing that dorsal expression of *Lhx5* was lost in the electroporated side of the embryo (Fig. 4G, H).

**Figure 5:**
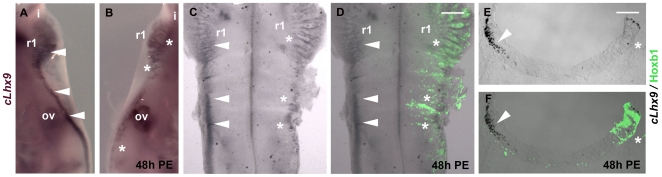
Expression of *Lhx5* in the chick hindbrain after *Hoxb1* gain of function experiments. (A – F) Expression of *Lhx9* in whole mount (A, B), flat mounted hindbrains (ventricular view) (C, D) and r1 transverse sections (E, F) of chick embryos electroporated at stage HH 10–11 and analyzed 48 h PE by *Lhx9 in situ* hybridization alone (A, B) or in combination with Hoxb1 immunofluorescence (C – F). Lxh9 is expressed in the mantle layer of dorsal r1 in a thick stripe that subsequently thins out along the rhombic lip of the rest of the hindbrain (arrowheads A, C, E, F). This expression is lost at sites of Hoxb1 ectopic expression (asterisks B, D, E, F). Scale bar corresponds to 300 µm in C, D and to 150 µm in E, F.

*Lhx9* is broadly expressed in the mouse developing CNS in the forebrain, midbrain, hindbrain and spinal cord. In the mouse, its levels of expression, as detected by RNA *in situ* hybridization, in the hindbrain were relatively low with no specific r4 pattern [43]. Using a chick *Lhx9 in situ* probe [39] we found that *cLhx9* is expressed in dorsal r1 and in a thin dorsal stripe in the developing chick hindbrain (arrowheads, Fig. 1A, C, D). Thus we choose to do our analysis in chick embryos by ectopically expressing *Hoxb1* in the developing hindbrain. Chick embryos were electroporated with *Hoxb1* expression vector at HH 10–11 and RNA *in situ* hybridization was performed 48h PE to detect c*Lhx9* expression. The expression of c*Lhx9* in the non-electroporated side was strong along the whole length of the hindbrain but, in the electroporated side, c*Lhx9* was down regulated in response to ectopic Hoxb1 expression. This was evident in whole mount embryos and flat mounted hindbrains (asterisks in Fig. 5B, C, D) and these findings were confirmed by cryosections (Fig. 5E, F).

Taken together these results showed that Hoxb1 represses expression of both *Lhx5* and *Lhx9* thus confirming the results of the microarray gene expression analysis in ES cell derived Hoxb1^−^ and Hoxb1^+^ NS cells.

## Discussion

The Hox patterning genes play diverse roles during embryo development in all three germ layer derivatives. An approach to understand their function was to compare the transcripteomes of wt tissue with tissues where Hox gene expression has been genetically manipulated [19], [20], [21], [22]. However, tissue heterogeneity, accumulation of long term effects that are not directly related to *Hox* gene function and functional redundancy among *Hox* genes limit the utility of this approach. Additionally, it is becoming increasingly evident that Hox activity is dependent upon extracellular signals and cellular context [5], [31], [44], [45], [46], [47], [48], [49], [50], [51]. Thus, to identify *Hox* target genes in a given cell specification process a model system recapitulating key aspects of this process could provide novel insights. We have shown that directed neural differentiation of mouse ES cells and inducible *Hoxb1* expression recapitulates key aspects of r4 neural specification [31]. Here we investigated whether this approach could be used to identify novel downstream effectors of *Hoxb1*.

Microarray gene expression analysis identified both induced and repressed genes in response to *Hoxb1* expression. Comparison of the effects of short term and long term *Hoxb1* induction showed that whereas *Hoxb1* acted as both activator and repressor of gene transcription in the short term, its long-term effects were mostly repressive suggesting that its fate selector function included active exclusion of alternative genetic programs. Strikingly, gene ontology (GO) analysis showed that up regulated and down regulated genes related to strictly distinct processes. The Hoxb1 repressing activity was directed primarily towards differentiation related processes whereas its activating functions were directed primarily towards early development, wnt and cell surface receptor linked signal transduction and cell-to-cell communication (Table 1). These results were consistent with the finding that *Hoxb1* expression delayed differentiation of ES derived NS cells in the absence of a mitogen and pinpointed likely effectors of these effects [31]. Thus *Hoxb1* plays a role in maintaining neural progenitor state and delaying differentiation. This does not rule out the possibility that *Hoxb1* may have distinct functions in post mitotic, maturing neural cells. A role in post mitotic maturation of motor neurons has been assigned to some members of the *Hox* family [30], [52], [53] and it is not understood whether distinct *Hox* genes are involved in either proliferating progenitors or post mitotic neural cells or both and to what extent. The approach described here offers a venue to address these issues.

Three other screens have been conducted to identify *Hoxb1* downstream effectors in r4 using tissue from mouse wt and Hoxb1^−/−^ hindbrains [19] or zebrafish wt and *Hoxb1a* knock down hindbrains [20] and by identifying the expression profiles of distinct mouse rhombomeres [36]. It is important to bear in mind that *Hox* gene activation in the mouse occurs around 7.5 dpc and the screens were performed at 9.5 or 10.5 dpc and, similarly, in zebrafish, *Hox* gene expression starts at around 10 hpf and the screen was conducted at 20 hpf. Thus there was ample time for multiple intermediate regulatory steps to take place and the observed readout was a combination of direct and indirect *Hox* targets, other patterning influences and co-regulated genes. In the screen based on ES derived NS these effects are minimized, due mainly to the absence of neighboring tissues, albeit not completely eliminated. In two of the studies selected genes were validated by corroborating changes in their expression profiles in wt and mutants [20], [36]. We have identified some, but not all, of these genes as well in our long induction scheme. Surprisingly, some of these genes were repressed in our screen rather than activated. A comparison of regulated genes in our long induction scheme revealed that about 10% (120 out of 1117) of them were also found regulated in the r4 of the Hoxb1^−/−^ mouse mutants [19]. Again, many of them were regulated in opposite directions (Table S3 and Table S4). An important difference between the methods followed previously and the approach described here is that the former combined cells of the ventricular and mantle layers at a time point when post mitotic neuronal cells abound whereas our approach relied on actively dividing neural progenitor cells representative of an earlier time point of development. This raises the intriguing possibility that some *Hoxb1* regulated genes switch from repressed to activated (and conversely) upon cell cycle exit. To *in vivo* validate some of our findings we corroborated the effects of Hoxb1 on the expression patterns of *CRABPI*, *CRABPII*, *Lhx5* and *Lhx9* using *in vivo* loss and gain of function models. *CRABPI*, *CRABPII* and *Lhx5* had a Hoxb1 dependent r4 specific expression pattern. It is worth noting that none of them was identified as such in the aforementioned screens underlining the sensitivity of the approach presented here.

Within the developing neural tube the diverse cellular distribution patterns of retinoid receptors and retinoid binding proteins indicates that it is necessary to fine-tune levels of RA signaling for the specification of diverse of neural subpopulations. CRABPI and II are located in the cytoplasm and bind RA, a key player in CNS pattern formation, neural specification and differentiation. *CRABP* expression was initially associated with structures that were more sensitive to excess of RA [54] and subsequent studies shed light in the function of these proteins. CRABPI participates in reducing the cellular RA response and associated differentiation by accelerating RA degradation [55], [56]. On the other hand, CRABPII acts as a ligand dependent coactivator of RAR translocating in the nucleus in the presence of RA thus facilitating its channeling to RAR and potentiating RA dependent transcriptional activation. [57], [58], [59]. Expression of both *CRABPI* and *II* was activated by *Hoxb1* in ES derived NS and these findings were validated in the mouse embryo since expression of both was down regulated in the r4 of Hoxb1^−/−^ reverting to expression patterns identical to those of r2. Intriguingly, *CRABPI* is up regulated whereas *CRABPII* is down regulated in the resident territory of r4 motor neurons suggesting that maturation and/or specification of this subpopulation needs particular shielding from RA exposure. Ectopic *Hoxb1* expression in r2 through timely supply of extraneous RA converts the r2 trigeminal motor neurons into r4 facial motor neurons [60], [61]. Conversely, loss-of function of Hoxb1 converts r4 facial motor neurons into trigeminal motor neurons [24], [25]. Thus RA is necessary for facial motor neuron specification acting as an upstream regulator of Hoxb1 [33], [34] and in turn, Hoxb1 fine-tunes RA availability through the regulation of *CRABPI* and *II* expression. However, further studies are needed to prove this hypothesis and establish whether *CRABPI/II* are direct Hoxb1 target genes. The localized expression of *RARa* in r4 and the localized expression of *Cyp1B1*, an atypical RA generating cytochrome, in the ventral r4 [36] lends further support for an important role of RA during the patterning of this territory. Both our screen and previous screens [19], [20], [36] have identified *RARa* as a Hoxb1 downstream target in r4. The ES derived NS cells are a mixture of different DV characters and this limits the detection capacity for markers that are exclusively expressed in distinct and narrow DV levels. This can be bypassed by dorsalising or ventralising these cells with appropriate DV morphogenetic signals [32]. It will be interesting to determine whether *Cyp1b1* is induced in shh treated ES derived Hoxb1^+^ NS cells as well.

The expression of several members of the LIM domain-containing subgroup of homeobox transcription factors (*Lhx* genes) was regulated by *Hoxb1* in ES derived NS cells. (Table S2). This subgroup is of considerable interest given that the LIM domain is a modified zinc finger domain that mediates interactions among transcription factors and their major, but not exclusive, role is patterning the CNS. *Lhx* genes define neuronal identity in a combinatorial manner and they control key aspects of neural cell fate decisions and neuronal differentiation including subtype identity and axonal guidance [35]. Thus they lay temporally downstream of the regionalization of the CNS controlled by *Hox* genes. In ES derived NS cells, *Hoxb1* postpones neural differentiation after mitogen withdrawal through the activation of the Notch signaling pathway [31]. The findings reported here suggest that Hoxb1 may do so partly by temporarily repressing expression of transcription factors such as *Lhx*. On the other hand, *Lhx8* was up regulated in ES derived NS cells by *Hoxb1* (Table S2) suggesting that Hox gene patterning activity may be exerted through both repression and activation of Lhx genes. Since Lhx8 is a key player in cholinergic neuron specification [62], Hoxb1 may participate in the specification of this subpopulation in the hindbrain. *Lhx5* and *Lhx9* are expressed broadly in the developing neural tube in specific subdomains [43]. Our findings suggest that *Hoxb1* can repress their expression but it is not yet known whether this is a direct effect. Nevertheless it does imply that Hox genes may act as upstream *Lhx* regulators in shaping their expression domains and thus participate in neuronal subtype specification.

The results of this study suggest that ES neural differentiation and inducible Hox gene expression can be used as a sensitive model system to address several important open issues pertaining to Hox gene function such as possible differential roles in ventricular and mantle zone neural cells, identify genome wide binding sites by chromatin immunoprecipitation studies, delineate the interactions of *Hox* genes and DV patterning signals in assigning neural identity and address the issue of specificity and functional overlap among different *Hox* genes.

## Supporting Information

Table S1 List of genes regulated by Hoxb1 induction in the long induction scheme as found by microarray gene expression profiling.
(XLS)

Table S2 List of genes regulated in both short (s) and long (l) induction schemes. Classification is according to primary GO process assignment. If not an assignment has been made genes are labelled as non-classified.
(XLS)

Table S3 List of of common genes induced in Hoxb1-/- r4 and also regulated by Hoxb1 in ES derived neural progenitors after long induction. At the top part of the list the observed regulation is in the same direction and after the space it is in the opposite direction.
(XLS)

Table S4 List of common genes repressed in Hoxb1-/- r4 and also regulated in ES derived neural progenitors after long induction. At the top part of the list the observed regulation is at the same direction and after the space it is in the opposite direction.
(XLS)

## References

[1] de Rosa R, Grenier JK, Andreeva T, Cook CE, Adoutte A (1999). Hox genes in brachiopods and priapulids and protostome
evolution.. Nature.

[2] McGinnis W, Krumlauf R (1992). Homeobox genes and axial patterning.. Cell.

[3] Wagmaister JA, Gleason JE, Eisenmann DM (2006). Transcriptional upregulation of the C. elegans Hox gene lin-39
during vulval cell fate specification.. Mech Dev.

[4] Hueber SD, Bezdan D, Henz SR, Blank M, Wu H (2007). Comparative analysis of Hox downstream genes in
Drosophila.. Development.

[5] Grienenberger A, Merabet S, Manak J, Iltis I, Fabre A (2003). Tgfbeta signaling acts on a Hox response element to confer
specificity and diversity to Hox protein function.. Development.

[6] Pöpperl H, Featherstone M (1993). Identification of a retinoic acid repsonse element upstream of
the murine *Hox-4.2* gene.. Mol Cell Biol.

[7] Kuziora MA, McGinnis W (1988). Autoregulation of a *Drosophila* homeotic selector
gene.. Cell.

[8] Gould A, Morrison A, Sproat G, White RA, Krumlauf R (1997). Positive cross-regulation and enhancer sharing: two mechanisms
for specifying overlapping Hox expression patterns.. Genes Dev.

[9] Garcia-Bellido A (1975). Genetic control of wing disc development in
Drosophila.. Ciba Found Symp.

[10] Yokouchi Y, Nakazato S, Yamamoto M, Goto Y, Kameda T (1995). Misexpression of *Hoxa-13* induces cartilage
homeotic transformation and changes cell adhesiveness in chick limb
buds.. Genes & Development.

[11] Bromleigh VC, Freedman LP (2000). p21 is a transcriptional target of HOXA10 in differentiating
myelomonocytic cells.. Genes Dev.

[12] Lohmann I, McGinnis N, Bodmer M, McGinnis W (2002). The Drosophila Hox gene deformed sculpts head morphology via
direct regulation of the apoptosis activator reaper.. Cell.

[13] Harris J, Honigberg L, Robinson N, Kenyon C (1996). Neuronal cell migration in C. elegans: regulation of Hox gene
expression and cell position.. Development.

[14] Samad OA, Geisen MJ, Caronia G, Varlet I, Zappavigna V (2004). Integration of anteroposterior and dorsoventral regulation of
Phox2b transcription in cranial motoneuron progenitors by homeodomain
proteins.. Development.

[15] Geisen MJ, Di Meglio T, Pasqualetti M, Ducret S, Brunet JF (2008). Hox paralog group 2 genes control the migration of mouse pontine
neurons through slit-robo signaling.. PLoS Biol.

[16] Chen J, Ruley HE (1998). An enhancer element in the EphA2 (Eck) gene sufficient for
rhombomere-specific expression is activated by HOXA1 and HOXB1 homeobox
proteins.. J Biol Chem.

[17] Pöpperl H, Bienz M, Studer M, Chan S, Aparicio S (1995). Segmental expression of *Hoxb1* is controlled by a
highly conserved autoregulatory loop dependent upon
*exd/Pbx*.. Cell.

[18] Svingen T, Tonissen KF (2006). Hox transcription factors and their elusive mammalian gene
targets.. Heredity.

[19] Tvrdik P, Capecchi MR (2006). Reversal of Hox1 gene subfunctionalization in the
mouse.. Dev Cell.

[20] Rohrschneider MR, Elsen GE, Prince VE (2007). Zebrafish Hoxb1a regulates multiple downstream genes including
prickle1b.. Dev Biol.

[21] Tkatchenko AV, Visconti RP, Shang L, Papenbrock T, Pruett ND (2001). Overexpression of Hoxc13 in differentiating keratinocytes results
in downregulation of a novel hair keratin gene cluster and
alopecia.. Development.

[22] Lei H, Wang H, Juan AH, Ruddle FH (2005). The identification of Hoxc8 target genes.. Proc Natl Acad Sci U S A.

[23] Diez del Corral R, Storey KG (2001). Markers in vertebrate neurogenesis.. Nat Rev Neurosci.

[24] Studer M, Lumsden A, Ariza-McNaughton L, Bradley A, Krumlauf R (1996). Altered segmental identity and abnormal migration of motor
neurons in mice lacking *Hoxb-1*.. Nature.

[25] Gavalas A, Ruhrberg C, Livet J, Henderson CE, Krumlauf R (2003). Neuronal defects in the hindbrain of Hoxa1, Hoxb1 and Hoxb2
mutants reflect regulatory interactions among these Hox
genes.. Development.

[26] Bell E, Wingate R, Lumsden A (1999). Homeotic transformation of rhombomere identity after localized
*Hoxb1* misexpression.. Science.

[27] Jacob J, Ferri AL, Milton C, Prin F, Pla P (2007). Transcriptional repression coordinates the temporal switch from
motor to serotonergic neurogenesis.. Nat Neurosci.

[28] Gaufo GO, Thomas KR, Capecchi MR (2003). Hox3 genes coordinate mechanisms of genetic suppression and
activation in the generation of branchial and somatic
motoneurons.. Development.

[29] Wu Y, Wang G, Scott SA, Capecchi MR (2008). Hoxc10 and Hoxd10 regulate mouse columnar, divisional and motor
pool identity of lumbar motoneurons.. Development.

[30] Dasen JS, Tice BC, Brenner-Morton S, Jessell TM (2005). A Hox regulatory network establishes motor neuron pool identity
and target-muscle connectivity.. Cell.

[31] Gouti M, Gavalas A (2008). Hoxb1 controls cell fate specification and proliferative capacity
of neural stem and progenitor cells.. Stem Cells.

[32] Gouti M, Briscoe J, Gavalas A (2011). Anterior Hox genes interact with components of the neural crest
specification network to induce neural crest fates.. Stem Cells.

[33] Marshall H, Studer M, Popperl H, Aparicio S, Kuroiwa A (1994). A conserved retinoic acid response element required for early
expression of the homeobox gene Hoxb-1.. Nature.

[34] Studer M, Pöpperl H, Marshall H, Kuroiwa A, Krumlauf R (1994). Role of a conserved retinoic acid response element in rhombomere
restriction of *Hoxb-1*.. Science.

[35] Dawid IB, Chitnis AB (2001). Lim homeobox genes and the CNS: a close
relationship.. Neuron.

[36] Chambers D, Wilson LJ, Alfonsi F, Hunter E, Saxena U (2009). Rhombomere-specific analysis reveals the repertoire of genetic
cues expressed across the developing hindbrain.. Neural Dev.

[37] Ruberte E, Friederich V, Morriss-Kay G, Chambon P (1992). Differential distribution patterns of CRABP-I and CRABP-II
transcripts during mouse embryogenesis.. Development.

[38] Sheng HZ, Bertuzzi S, Chiang C, Shawlot W, Taira M (1997). Expression of murine Lhx5 suggests a role in specifying the
forebrain.. Dev Dyn.

[39] Sun X, Saitsu H, Shiota K, Ishibashi M (2008). Expression dynamics of the LIM-homeobox genes, Lhx1 and Lhx9, in
the diencephalon during chick development.. Int J Dev Biol.

[40] Varela-Echavarria A, Pfaff SL, Guthrie S (1996). Differential expression of LIM homeobox genes among motor neuron
subpopulations in the developing chick brain stem.. Mol Cell Neurosci.

[41] Stamataki D, Ulloa F, Tsoni SV, Mynett A, Briscoe J (2005). A gradient of Gli activity mediates graded Sonic Hedgehog
signaling in the neural tube.. Genes Dev.

[42] Gavalas A, Trainor P, Ariza-McNaughton L, Krumlauf R (2001). Synergy between Hoxa1 and Hoxb1: the relationship between arch
patterning and the generation of cranial neural crest.. Development.

[43] Gray PA, Fu H, Luo P, Zhao Q, Yu J (2004). Mouse brain organization revealed through direct genome-scale TF
expression analysis.. Science.

[44] Walsh CM, Carroll SB (2007). Collaboration between Smads and a Hox protein in target gene
repression.. Development.

[45] Wang N, Kim HG, Cotta CV, Wan M, Tang Y (2006). TGFbeta/BMP inhibits the bone marrow transformation capability of
Hoxa9 by repressing its DNA-binding ability.. EMBO J.

[46] Arata Y, Kouike H, Zhang Y, Herman MA, Okano H (2006). Wnt signaling and a Hox protein cooperatively regulate psa-3/Meis
to determine daughter cell fate after asymmetric cell division in C.
elegans.. Dev Cell.

[47] Joulia L, Deutsch J, Bourbon HM, Cribbs DL (2006). The specification of a highly derived arthropod appendage, the
Drosophila labial palps, requires the joint action of selectors and
signaling pathways.. Dev Genes Evol.

[48] Marty T, Vigano MA, Ribeiro C, Nussbaumer U, Grieder NC (2001). A HOX complex, a repressor element and a 50 bp sequence confer
regional specificity to a DPP-responsive enhancer.. Development.

[49] Taghli-Lamallem O, Hsia C, Ronshaugen M, McGinnis W (2008). Context-dependent regulation of Hox protein functions by CK2
phosphorylation sites.. Dev Genes Evol.

[50] Hueber SD, Lohmann I (2008). Shaping segments: Hox gene function in the genomic
age.. Bioessays.

[51] Mann RS, Lelli KM, Joshi R (2009). Hox specificity unique roles for cofactors and
collaborators.. Curr Top Dev Biol.

[52] Dasen JS, De Camilli A, Wang B, Tucker PW, Jessell TM (2008). Hox repertoires for motor neuron diversity and connectivity gated
by a single accessory factor, FoxP1.. Cell.

[53] Miguel-Aliaga I, Thor S (2004). Segment-specific prevention of pioneer neuron apoptosis by
cell-autonomous, postmitotic Hox gene activity.. Development.

[54] Vaessen M-J, Meijers J, Bootsma D, Van Kessel A (1990). The cellular retinoic-acid-binding protein is expressed in
tissues associated with retinoic-acid-induced malformations.. Development.

[55] Boylan JF, Gudas LJ (1992). The level of CRABP-I expression influences the amounts and types
of all-trans-retinoic acid metabolites in F9 teratocarcinoma stem
cells.. J Biol Chem.

[56] Boylan JF, Gudas LJ (1991). Overexpression of the cellular retinoic acid binding protein-I
(CRABP-I) results in a reduction in differentiation-specific gene expression
in F9 teratocarcinoma cells.. J Cell Biol.

[57] Dong D, Ruuska SE, Levinthal DJ, Noy N (1999). Distinct roles for cellular retinoic acid-binding proteins I and
II in regulating signaling by retinoic acid.. J Biol Chem.

[58] Budhu A, Gillilan R, Noy N (2001). Localization of the RAR interaction domain of cellular retinoic
acid binding protein-II.. J Mol Biol.

[59] Delva L, Cornic M, Balitrand N, Guidez F, Miclea JM (1993). Resistance to all-trans retinoic acid (ATRA) therapy in relapsing
acute promyelocytic leukemia: study of in vitro ATRA sensitivity and
cellular retinoic acid binding protein levels in leukemic
cells.. Blood.

[60] Marshall H, Nonchev S, Sham MH, Muchamore I, Lumsden A (1992). Retinoic acid alters hindbrain Hox code and induces
transformation of rhombomeres 2/3 into a 4/5 identity.. Nature.

[61] Kessel M (1993). Reversal of axonal pathways from rhombomere 3 correlates with
extra Hox expression domains.. Neuron.

[62] Zhao Y, Marin O, Hermesz E, Powell A, Flames N (2003). The LIM-homeobox gene Lhx8 is required for the development of
many cholinergic neurons in the mouse forebrain.. Proc Natl Acad Sci U S A.

